# Assessing the level of awareness of sports science students about the causes of marine trauma

**Published:** 2022-01

**Authors:** Javad Moghadasi, Leila Keikavoosi-Arani, Elham Ehsani-Chimeh

**Affiliations:** ^*a*^ Ph.D. in Higher Education Management, Tehran Science and Research Branch, Islamic Azad University, Tehran, Iran.; ^*b*^ Assistant Professor of Healthcare Services Management, Department of Healthcare Services Management, School of Health, Alborz University of Medical Sciences, Karaj, Iran.; ^*c*^ Assistant Professor of Healthcare Services Management, National Institute for Health Research, Tehran University of Medical Sciences, Tehran, Iran.

## Abstract

**Background:**

Marine trauma threatens the lives of many people in Iran and other countries every year. It affects a majority of the young population who are in a critical period of life to be effective for the development of their society. Increasing awareness can have a positive effect on reducing these injuries. This study aimed to assess the awareness of sports science students about the factors that cause marine trauma.

**Methods:**

The statistical population of this descriptive survey was all sports science students in the Islamic Azad University of Science and Research in 2021 (N = 325). The sample size based on Morgan's table and Cochran's formula calculation was 176 people who were selected by stratified sampling method. The data collection tool was a two-part researcher-made questionnaire including demographic information and questions. Data analysis was performed using SPSS21 software and t-test, Wilcoxon, Kruskal-Wallis, and one-way analysis of variance.

**Results:**

The knowledge of the majority of students (62.5%) about marine trauma was below average. The lowest score (1.86) belonged to "awareness of trauma caused by strong winds and storms" and the highest score (4.76) was related to "awareness of injuries caused by the bite of marine animals (sharks, etc."). There was no statistically significant relationship between gender and awareness of marine trauma. There was a significant relationship between age, education, and experience in swimming with the level of awareness of marine trauma in some components.

**Conclusions:**

The majority of sports science students had poor awareness about the causes of marine trauma, which shows the need to pay attention to increasing the level of awareness of these students.

**Keywords:**

Marine, Trauma, Sports Science, Education

